# Machine learning for automating subjective clinical assessment of gait impairment in people with acquired brain injury – a comparison of an image extraction and classification system to expert scoring

**DOI:** 10.1186/s12984-024-01406-w

**Published:** 2024-07-23

**Authors:** Ashleigh Mobbs, Michelle Kahn, Gavin Williams, Benjamin F. Mentiplay, Yong-Hao Pua, Ross A. Clark

**Affiliations:** 1 School of Health, University of the Sunshine Coast, Sippy Downs, QLD Australia; 2 Department of Physiotherapy, Epworth Healthcare, Richmond, VIC Australia; 3 School of Health Sciences, University of Melbourne, Parkville, VIC Australia; 4 School of Allied Health, Human Services and Sport, La Trobe University, Bundoora, VIC Australia; 5 Department of Physiotherapy, Singapore General Hospital, Singapore, Singapore; 6 Duke-National University of Singapore Medical School, Singapore, Singapore

## Abstract

**Background:**

Walking impairment is a common disability post acquired brain injury (ABI), with visually evident arm movement abnormality identified as negatively impacting a multitude of psychological factors. The International Classification of Functioning, Disability and Health (ICF) qualifiers scale has been used to subjectively assess arm movement abnormality, showing strong intra-rater and test-retest reliability, however, only moderate inter-rater reliability. This impacts clinical utility, limiting its use as a measurement tool. To both automate the analysis and overcome these errors, the primary aim of this study was to evaluate the ability of a novel two-level machine learning model to assess arm movement abnormality during walking in people with ABI.

**Methods:**

Frontal plane gait videos were used to train four networks with 50%, 75%, 90%, and 100% of participants (ABI: *n* = 42, healthy controls: *n* = 34) to automatically identify anatomical landmarks using DeepLabCut^™^ and calculate two-dimensional kinematic joint angles. Assessment scores from three experienced neurorehabilitation clinicians were used with these joint angles to train random forest networks with nested cross-validation to predict assessor scores for all videos. Agreement between unseen participant (i.e. test group participants that were not used to train the model) predictions and each individual assessor’s scores were compared using quadratic weighted kappa. One sample t-tests (to determine over/underprediction against clinician ratings) and one-way ANOVA (to determine differences between networks) were applied to the four networks.

**Results:**

The machine learning predictions have similar agreement to experienced human assessors, with no statistically significant (*p* < 0.05) difference for any match contingency. There was no statistically significant difference between the predictions from the four networks (F = 0.119; *p* = 0.949). The four networks did however under-predict scores with small effect sizes (p range = 0.007 to 0.040; Cohen’s d range = 0.156 to 0.217).

**Conclusions:**

This study demonstrated that machine learning can perform similarly to experienced clinicians when subjectively assessing arm movement abnormality in people with ABI. The relatively small sample size may have resulted in under-prediction of some scores, albeit with small effect sizes. Studies with larger sample sizes that objectively and automatically assess dynamic movement in both local and telerehabilitation assessments, for example using smartphones and edge-based machine learning, to reduce measurement error and healthcare access inequality are needed.

## Introduction

In 2019, global incidents of acquired brain injury (ABI), specifically stroke and traumatic brain injury, were estimated to be 80 and 69 million people respectively [1, 2]. Walking impairment is a common physical disability post moderate to severe ABI [3, 4] affecting the legs, trunk and upper limbs, which limits participation in activities of daily living, adversely impacting quality of life [5, 6]. These visible deficits may create aesthetic issues, adding to the stigma of disability, and negatively impacting body image, self-esteem, mental health and social integration [7–9].

The criterion reference method for objective walking assessment is three-dimensional motion analysis [10]; however, it is a resource intensive endeavour, with cost and marker setup being a major barrier to clinical application. Cimolin and Galli [11] also outlined the limitation of clinicians needing to interpret the vast amount of data. This leads to concerns regarding pragmatic application in a clinical setting. Other systems such as inertial measurement units hold promise for obtaining kinematic data during functional tasks in clinical settings, but they too have extensive setup and calibration times in addition to infection control issues related to sanitising them between patients that often preclude their use in routine practice [12].

Physiotherapists are believed to be skilled in accurate observational assessment of movement [13]. For this reason, and because of its excellent clinical utility, subjective rating via visual observation is a frequently employed clinical assessment method and the most common tool utilised for clinical gait assessment [14, 15]. Prior work has therefore evaluated the application of the International Classification of Functioning, Disability and Health Framework (ICF) Qualifiers Scale [16] for subjective assessment of arm movement abnormality during walking in people with ABI [17]. Whilst this assessment method has been shown to have strong intra-rater and test-retest reliability, its inter-rater reliability was only moderate [17]. For clinical practice settings where assessors may differ over time, this attenuates the capacity to detect clinically relevant, but potentially subtle, changes in joint movement abnormality, thereby limiting clinical application.

Machine learning predictive algorithms are often applied to look for patterns in data and yield valuable outcome measurements and classifications [18, 19]. Machine learning has seen increased application in healthcare from prediction tasks to image classification, often outperforming humans [19]. Recent advancements in marker-less pose estimation software, such as DeepLabCut^™^, allow the training of networks that can extract joint positions from video frames [20]. These joint positions in a time series may be used as data to provide features for a predictive machine learning model that can assess functional tasks such as walking and other dynamic movements. Where machine learning may be particularly useful is in the classification of subjective assessments, which usually relies on a clinician visually inspecting a movement and rating it against their own interpretation of the scoring system. For example, recent studies have used DeepLabCut^™^ generated pose estimation data to predict (1) subjective scores on a general movement assessment in children at high risk of cerebral palsy [21], and (2) lying or sitting arm and leg dystonia in children living with cerebral palsy [22]. This has potential for inter-rater reliability error, which is particularly concerning if these data are being used to inform clinical practice and treatment decision making. Machine learning algorithms could however be trained using a composite or average of several assessors, theoretically enabling the resulting algorithm to provide a score analogous to the consensus results this group of assessors would derive even when exposed to data it has not seen before. If enough raters are used in the training of the model, the law of large numbers may eventuate in that the true value would be detected and outlying results, which would be evident if enough individual assessors were performing these assessments, would be removed. Compared to other instrumented methods such as marker or inertial measurement unit-based 3D gait analysis it has the potential to be performed and interpreted in close to real-time, and without sensors or markers placed on the person, enhancing its efficiency and potential clinical utility by overcoming the previously described limitations of these methods such as infection control. This could provide further benefits for in-person treatment settings, by reducing the assessor burden, improving accuracy, and addressing an identified telehealth issue whereby clinicians feel their scope for assessment is reduced [23].

To our knowledge there has been limited application of this machine learning methodology in the context of complex motor movements for predicting subjective gait assessment scores. Three recent studies have applied wearable sensors and/or an RGB-D (red, green, blue – depth, for example the Microsoft Kinect) or standard computer cameras to model machine learning algorithms to score relatively stationary upper body tasks such as Fugl-Meyer and Unified Parkinson’s Disease Rating Scale, and finger tapping assessments [24–26]. These studies compared their machine learning model results against either purely subjective scoring [24, 25] or a combination of subjective and wearable sensor-based scoring [26]. Additionally, a small convenience sample (*n* = 8) study used DeepLabCut^™^ to identify anatomical landmark data and neural networks to predict lower limb gait parameters and compare these data to a clinical system for spatiotemporal gait analysis (GAITRite^®^) [27]. Other studies have used machine learning to classify gait patterns based on self-reported measures, such as depression [28], or to identify simulated gait impairment in healthy people [29]. However, none of these studies attempted to quantify kinematic marker-less upper limb movement collected in a clinical population, during a dynamic task as complex as walking, and use these data to predict the results of a subjective rating scale performed by experienced clinicians. Therefore, the primary aim of this study was to examine how well a two-level machine learning model can assess arm movement abnormality during walking in people with ABI. This model consists firstly of an image analysis program that extracts relevant anatomical landmarks, and secondly a program that uses these anatomical landmarks to provide a subjective rating score. We hypothesise that the machine learning algorithm will predict arm movement abnormality scores during gait with similar agreement and accuracy as a trained experienced clinician. A secondary aim was to examine how the proportion of videos used to train versus test the algorithm impacted the accuracy, with four variations of train/test proportions (50%; 75%; 90%; 100%). For this, we hypothesised that increasing the relative proportion of trained videos would increase the accuracy of the prediction.

## Methods

### Human ethics and consent to participate declaration

This study included participants with adult-onset ABI (e.g. stroke and traumatic brain injury) from a previous cross-sectional observational study [17]. Ethics approval was received from Human Research Ethics Committees of Epworth Healthcare (approval number 648 − 14) and the University of the Sunshine Coast (approval number S/17/1006) in accordance with the Declaration of Helsinki.

### Participants

The experimental group demonstrated an effort dependent arm movement abnormality in their hemiplegic or affected upper limb during walking. Participants were a convenience sample of individuals (*n* = 42). They were recruited from a brain injury rehabilitation centre and private practice neurological-based physiotherapy clinics in Melbourne, Australia. All participants provided written informed consent before assessment. Participants were > 18 years of age and were required to walk > 10 m barefoot, with no walking aids or hands-on assistance. Participants were excluded if they did not understand the English instructions, were pregnant, medically unstable, had significant cognitive impairments, or had physical deficits preventing assessment completion.

A convenience sample of healthy controls (HCs) was recruited from the healthcare network of staff, family, and friends (*n* = 34). Controls were included if they were more than 18 years of age and had no comorbidities that impacted their upper limbs or capacity to walk. Both groups were required to walk on a 10 m walkway at a self-selected and fastest possible safe walking speed. Two trials of each task were recorded and analysed.

### Procedure

A Microsoft Kinect V2 camera was placed 0.5 m from the end of the walkway to record the RGB images (resolution 1920 × 1080) of participants in the frontal plane, for a total walkway length of 10.5 m. The ICF qualifiers scale was used by the expert assessors to quantify arm movement abnormality during walking. Additionally, the peak elbow flexion during walking was estimated visually by the assessors. Assessment was conducted by three neurological physiotherapists with > 15 years’ experience (therapist 1: 16 years, therapist 2: 33 years and therapist 3: 19 years). Assessors were provided with a lay definition of the rating categories and the video order was randomised. Physiotherapists observed the videos at standard playback speed with no rewinding in one 4-hour session with a 30-minute break using a reference sheet and a spreadsheet to input values. Before assessing videos, a 10-minute overview of arm movement abnormality during walking was provided with project rationale, along with two familiarisation videos to practice applying the scale. Videos were assessed and scored for an arm movement abnormality during walking, with 159 videos for the ABI group across *n* = 42 participants, and 5 videos for the HCs across a randomly sampled *n* = 5 participants. Only 5 HC videos were included in the subjective analysis to reduce biasing results, as these videos would by definition be scored as unimpaired and hence including the entire dataset would artificially inflate our validity findings. Conversely, all HC videos were used in training the first level of the machine learning algorithm, which was designed to detect anatomical landmarks.

### First machine learning level: anatomical landmark network development

**Fig. 1:**
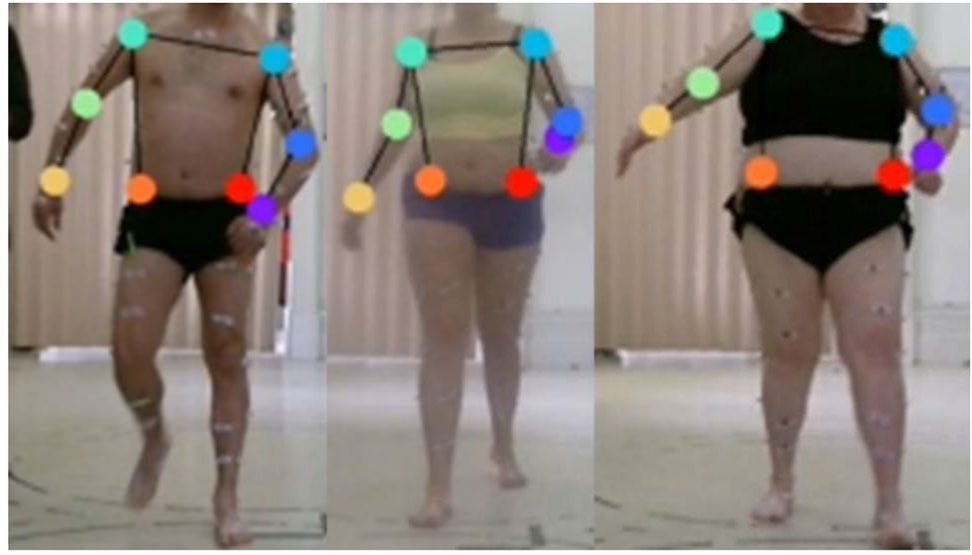
Examples of labelled videos with anatomical landmarks, resulting from applying the trained networks to label videos. The anatomical landmarks identified were approximations of the shoulder, elbow and wrist centres on each arm, and the anterior superior iliac spine ridges on both sides of the pelvis. Note the physical markers that can be seen in the images were not used in this analysis

This study utilised an open-source software package (DeepLabCut^™^) applied to compute 2D and 3D pose estimates [20]. DeepLabCut^™^ was applied to select training data frames, label data frames, and generate training data for label positions over time. Videos were selected in numerical participant order selecting 50% (ABI: *n* = 21, HCs: *n* = 17), 75% (ABI: *n* = 32, HCs: *n* = 26), 90% (ABI: *n* = 38, HCs: *n* = 31), or 100% (ABI: *n* = 42, HCs: *n* = 34) of the participants that were used to train the network for each group. Only one video was selected randomly for each participant between the available videos. Still images were selected using the uniform method within DeepLabCut^™^ to select frames, labelling 20 frames per video. Bilateral joint label locations of shoulders, elbows, wrists, and hips were applied to each extracted frame (Fig. 1). Selection of training iterations was informed by Mathis, Mamidanna [20], with the DeepLabCut^™^ settings being 500,000 training iterations, using a 101 layer deep convolutional neural network (setting: resnet101), with no repeated shuffles (setting: shuffle = 1).

### Anatomical landmark extraction

Anatomical landmark positions were then imported into a customised software package to extract the maximum, minimum, mean, standard deviation, skew, and kurtosis of the frontal plane elbow and shoulder angles of each limb. A recent study using 3D motion analysis demonstrated that the shoulder abduction and elbow flexion were the largest weighted variables influencing arm movement abnormality during walking, therefore, these measures were selected as the target features for this assessment [30]. Frontal plane elbow angle was calculated as the angle between the longitudinal axes of the wrist to elbow segment, and the elbow to shoulder segment. The frontal plane shoulder angle was calculated as the angle between the longitudinal axes of the elbow to shoulder segment, and a line running vertically between the midpoint of the two hip landmarks and the two shoulder landmarks. This created a longitudinal axis running approximately through the sacrum and sternum on the anterior aspect of the body. Data were automatically trimmed to extract movements associated with gait, and exclude stationary time (start of the trial) and missing markers due to the person being too close to the camera (end of the trial). The former was achieved by creating an algorithm that identified when the pixel distance between any of the hip or shoulder markers increased, indicating that the image of the person was larger in the frame and hence they were closer to the camera. The latter was achieved by excluding all data once any of the identified anatomical landmarks were lost from the image for more than two consecutive frames. These kinematic traces for individual anatomical landmarks were three-point median filtered to reduce error associated with single frame landmark error. No filtering of the joint angle traces was performed.

### Second machine learning level: predictive algorithm development

Whilst all recorded videos for all participants were used for anatomical landmark training, as stated previously not all HCs videos were used for the second stage of algorithm development (i.e. subjective score prediction). Only 5 HCs videos were scored using the subjective rating scale, as they would by nature score very well, and hence including all healthy participants would skew the data. Therefore, each of the four trained networks were applied to all videos that had been assessed and scored for an arm movement abnormality during walking (ABI: 159 videos, HCs: 5 videos).

The maximum, minimum, mean, standard deviation, skew, and kurtosis for the bilateral elbow and shoulder was applied alongside the median subjective score of arm movement abnormality during walking for the three assessors across videos. Importantly, the median was selected as it allowed the value from the three assessors to maintain an ordinal value, appropriate for classification and analogous to the nature of the assessment. Spyder (version 4.2.5) was used within an Anaconda environment (version 4.11.0) using version 3.7.9 of Python. The Scikit-learn module (version 1.0.2) was applied to fit classification models, scale data, and perform K-fold validation [31]. Spreadsheets were imported into Spyder with features scaled using the StandardScaler to remove the mean and scale to unit variance. During pilot testing we compared a variety of different classification models (random forest, K-nearest neighbours, support vector, logistic, and decision tree) to choose an appropriate model. Subjectively we observed that the random forest classification was at least as good overall as the other methods and given that it has the advantage of providing the weighting that each variable contributes to the predictive algorithm, was subsequently chosen to represent all networks.

To validate and assess the models, K-fold validation was applied to each classification model. K-fold validation discussed by Zhang and Yang [32] involves splitting data into k = 5 equal outer folds where 1 outer fold is held as a “test set” and the remaining four folds are used as a “training set” to train a model, this process is repeated for each outer fold to be the test set. The accuracy was calculated for each test set with the accuracy of the test sets taken across each classification model.

Using a validation method introduced by Varma and Simon [33], hyperparameters for each classification model were tuned by applying inner cross-validation loops to each training set of the outer cross-validation loop. Nested cross validation, whilst not frequently used due to being computationally expensive, was used to optimise hyperparameters and deemed appropriate for our relatively small dataset [34]. Our model used five folds for cross-validation.

**Fig. 2:**
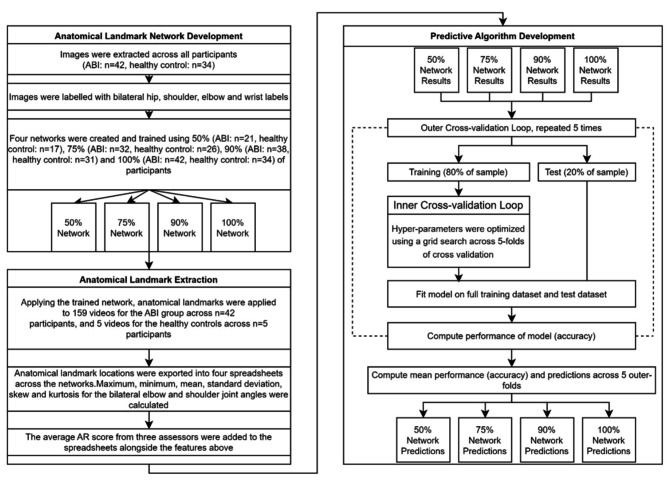
Analysis pipeline describing the steps applied in sequential order for the first and second layer of machine learning to develop network predictions for each of the four networks (50%, 75%, 90%, 100%)

Outer and inner cross validation processes were automated using Python to calculate parameters with respect to accuracy across all inner loops. Outer cross-validation was also automated with Python code reporting the average accuracy for each fold and average accuracy across the five folds for each classification model. The trained model was then applied to predict the scores for each video in the dataset across all networks (50%, 75%, 90%, 100%), outputting an array of predicted scores for each network. An overview of the overall process of the machine learning implementation is provided in Fig. 2.

### Statistical analysis

The predicted scores were imported and compared to the median and individual assessor scores with accuracy ($$\frac{Number of correctly classifed assessments}{Total number of assessments}*100$$) and mean square error (MSE) ($$\frac{1}{n}\sum\nolimits_{i = 1}^n {{{\left( {{\rm{Y}}i - {\rm{\hat Y}}i} \right)}^2}}$$), where *n* is the number of data points, $$\text{Y}i$$ is the observed value and $${\rm{\hat Y}}i$$ is the predicted value. Accuracy and MSE were calculated between the median of the three assessors and the machine learning networks (50% vs. median prediction, 75% vs. median prediction, 90% vs. median prediction, 100% vs. median prediction) and reported. Predicted scores were subtracted from the median assessor score. Absolute values from this procedure were tallied and reported to show the distribution for the prediction vs. assessors.

The predicted values for each network were subtracted from the median score between assessors and plotted using histograms to visualise the distribution. To assess the variance and performance of the four networks, a one-way ANOVA test was applied. One-sample t-tests were also performed for each network to examine if the network had a tendency to over- or under-predict values. Probability values of < 5% (i.e. *p* < 0.05) were considered to be significant for evaluation. An agreement matrix was developed by applying a quadratic weighted Cohen’s kappa [35]. Unseen participants (i.e. test group participants that were not used to train the model) were used from the 50% network, comparing between individual experienced assessors and the machine learning predictions. Additionally, a contingency table was created after all data for the 50% machine learning network and the median of the assessor scores were converted to (1) Impaired: moderate or higher impairment (score of 2 or greater) or (2) Unimpaired: minimal to no impairment (score of 0 or 1), which has been done in prior research using ICF scoring to convert the scale to a binary outcome [36]. From these data the recall ($$\frac{TP}{TP+FN}$$), precision ($$\frac{TP}{TP+FP}$$), accuracy ($$\frac{TP+TN}{TP+FP+TN+FN}$$), and summary F1 ($$\frac{2*precision*recall }{precision+recall}$$) scores were calculated, where TP = true positive, TN = true negative, FP = false positive and FN = false negative. All analyses were performed using Excel and Python. When assessing differences between predictions for participants who were seen or unseen by the DeepLabCut^™^ network a histogram was used. The predictions were filtered by seen vs. unseen and subtracted from the median assessor score for the appropriate video file.

## Results

76 people were recruited for walking trials, 42 had an ABI (25 with stroke, 15 with traumatic brain injury, and 2 with prior cerebral neurosurgery, 34 were HCs. Descriptive data are reported in Table 1, with participants details further described in Kahn, Clark [17]. Amongst the group of people with ABI they were predominately male (26/42), conversely, within the HCs they were predominantly female (21/34). People within the ABI group had a mean time post injury of 6.2 years (± 5.7 years).

**Table 1:** Study participant characteristics between the ABI (*n* = 42) and the HCs (*n* = 34)

Characteristics	Subjects with ABI (*n* = 42)		HCs (*n* = 34)	
	Descriptive	Range	Descriptive	Range
Sex (male/female)	26/16	-	13/21	
Age (years)	48 ± 17	20 to 84	37 ± 15	21 to 78
Weight (kg)	80 ± 16	47 to 131	70 ± 13	45 to 103
Height (cm)	172 ± 8	155 to 190	170 ± 10	150 to 188
Injury Type (n)	TBI (15) CVA (25) NS (2)	-		
Time Post Injury (yrs)	6.2 ± 5.7	0.2 to 40.4		
Hemiplegic side (L/R)	26/16	-		

**Fig. 3:**
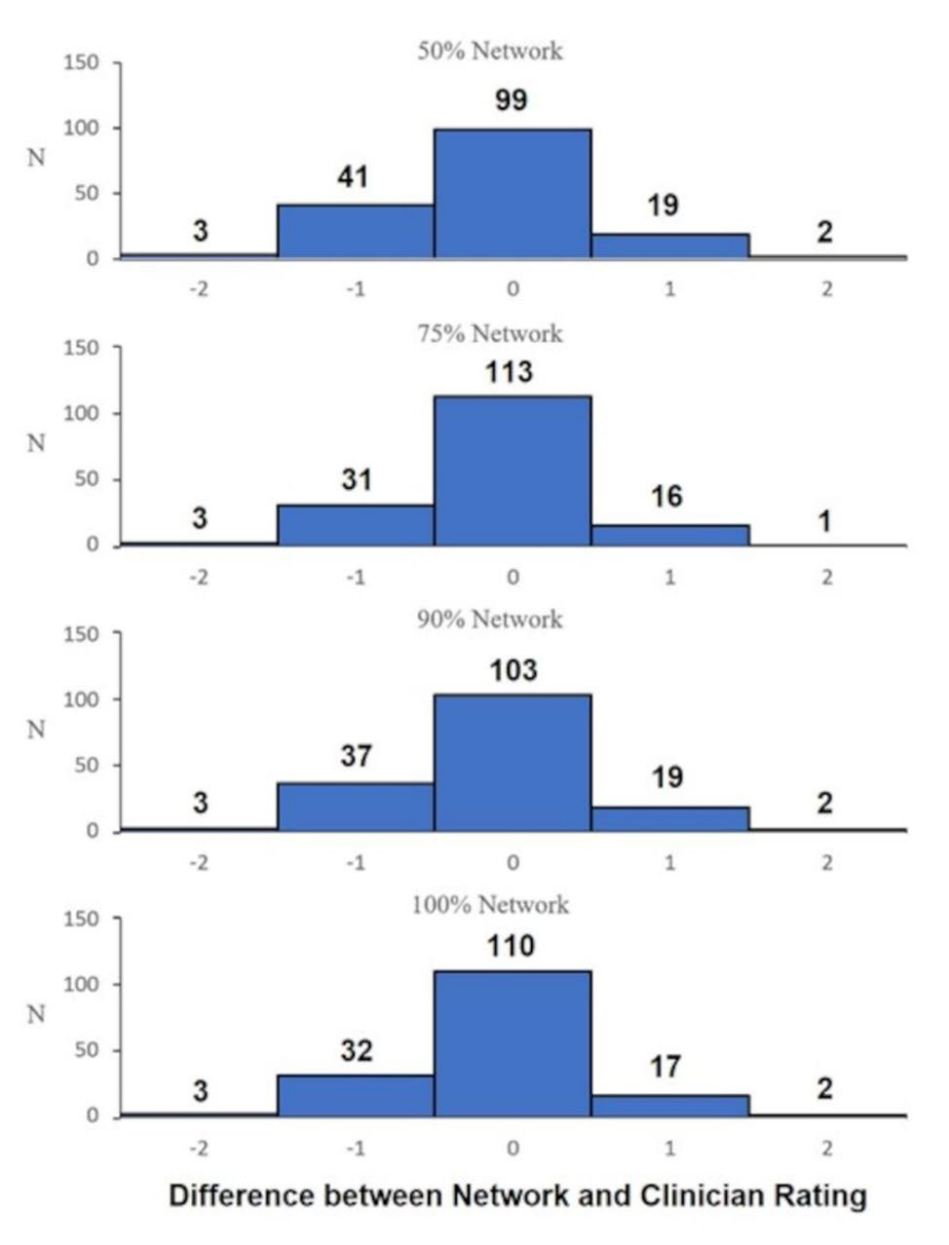
Histogram illustrating the distribution of predicted scores subtracting median assessor score for each of the four networks

For the first part of the algorithm training (i.e., anatomical landmark identification) train and test errors ranged between 1.35 and 4.15 pixels across all networks (50%,75%,90%,100%) applied to the 1920 × 1080 video frames. A one-way ANOVA between the networks (50%, 75%, 90%, 100%) found that there were no statistically significant differences between the predictions (F = 0.119; *p* = 0.949). The distribution between the four networks subtracting the median assessor score are presented in Fig. 3, qualitatively demonstrating that all networks are similarly distributed. One sample t-tests for each network revealed that there was a statistically significant difference to 0 (p range = 0.007 to 0.04; Cohen’s d range = 0.156 to 0.217), indicating that the models tended to under-predict subjective scores, however, with small effect size [37].

**Fig. 4:**
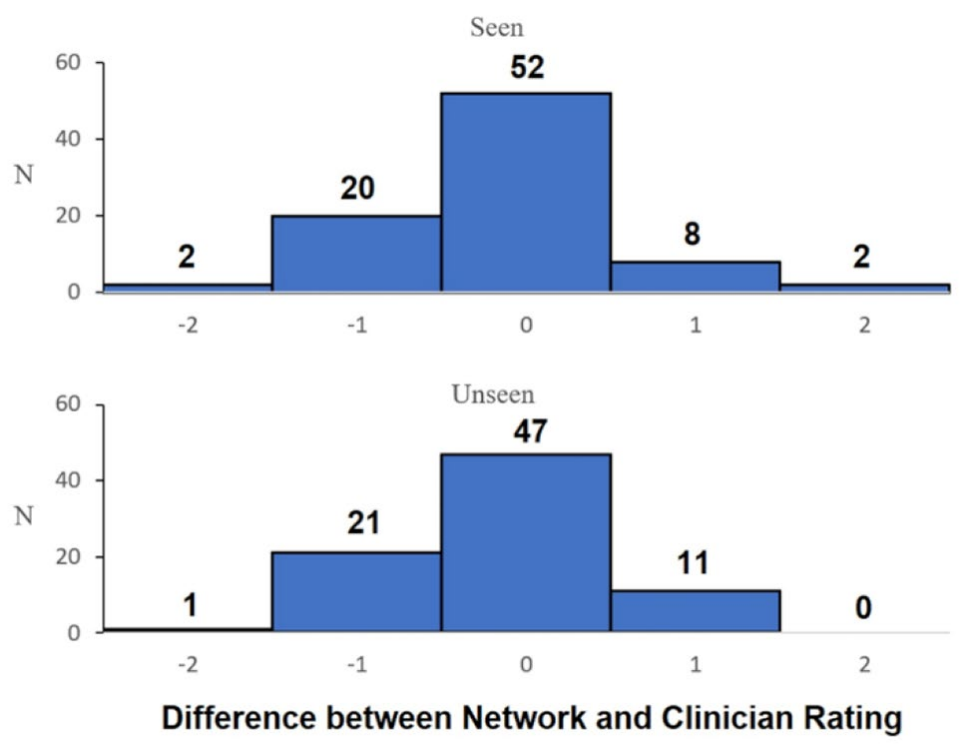
Histogram comparing the difference in the median assessor score between the seen and unseen participants for the 50% (right: ABI: *n* = 21, HCs: *n* = 17) network. 75%, 90% and 100% networks were omitted due to the corresponding small sample size of the unseen participant group

Figure 4 displays a histogram comparing the predicted scores of seen vs. unseen participants. Qualitatively, this highlights that there is no discernible difference in the predictive algorithm’s capacity to predict subjective scores between the seen and unseen condition.

As shown in Table 2 with respect to the difference between scores, the four machine learning groups had similar results to each other when comparing the accuracy and absolute difference between scores, with no discernible pattern related to percentage of videos used for training. Amongst the unseen comparisons, 50% vs. assessor 1 and 50% vs. assessor 2 appeared to marginally underperform when compared to comparisons between assessors (assessor 1 vs. assessor 2, assessor 1 vs. assessor 3, assessor 2 vs. assessor 3). However, the 50% network performed similarly when compared to assessor 2 with similar distributions evident between assessors (assessor 1 vs. assessor 2 and assessor 2 vs. assessor 3).

**Table 2:** The accuracy, mean square error (MSE) and difference between scores for the predicted scores for the four networks compared to the mean of the three assessors. Also shown is the difference between scores between each individual assessor and the 50% network’s predictions for only the unseen participants

Comparison	Accuracy (%)	MSE	Absolute difference between scores			
			0	1	2	3
*All data included*						
50% vs. A_med_	60.4	0.49	99	60	5	0
75% vs. A_med_	68.9	0.38	113	47	4	0
90% vs. A_med_	62.8	0.46	103	56	5	0
100% vs. A_med_	67.1	0.42	110	49	5	0
*Unseen data only*						
A1 vs. A2 (unseen)	57.5	0.66	46	31	1	2
A1 vs. A3 (unseen)	66.3	0.38	53	26	1	0
A2 vs. A3 (unseen)	67.5	0.36	54	25	1	0
50% vs. A_med_ (unseen)	58.8	0.45	47	32	1	0
50% vs. A1 (unseen)	47.5	0.68	38	38	4	0
50% vs. A2 (unseen)	52.5	0.51	42	37	1	0
50% vs. A3 (unseen)	63.8	0.40	51	28	1	0

A_med_ = median value of the three Assessors; A1 = Assessor 1; A2 = Assessor 2; A3 = Assessor 3;

Table 3 displays a matrix comparing the inter-rater agreement between each assessor and the 50% unseen network. This table highlights that within each of the three match contingencies there is no statistically significant (*p* < 0.05) difference between the predictions of the experienced clinicians and the 50% unseen network predictions.

**Table 3:** Agreement matrix applying a quadratic weighted kappa with standard error between each of the assessors and 50% machine learning network for unseen participants only

	50% Unseen Network Predictions	Assessor 3	Assessor 2
Assessor 1	0.41 ± 0.11	0.71 ± 0.19	0.58 ± 0.18
Assessor 2	0.55 ± 0.13	0.73 ± 0.11	-
Assessor 3	0.59 ± 0.17	-	-

Table 4 displays a contingency table after all data for the 50% machine learning network and the median of the assessor scores were converted to binary impaired/unimpaired. This showed that the model had a very high level of recall (89%), precision (88%), and accuracy (84%), and the summary F1 score was also very high (0.88).

**Table 4:** Contingency table showing the agreement between the 50% machine learning network and the median of the assessors scores after binary conversion of the scoring to impaired (score *≥* 2) or unimpaired (score *≤* 1)

		Assessor Median	
		Impaired	Unimpaired
50% ML	Impaired	49	7
	Unimpaired	6	18

## Discussion

We have demonstrated that, when trained and implemented using the methodology described in this study, machine learning predictions can result in similar agreement to human assessors for the relatively complex task of assigning a subjective rating of severity score to a video of someone walking with arm movement abnormality. For example, our 50% network was able to classify the 0–4 scale scores with 60.4% and 58.8% accuracy for the total data and unseen data only respectively when compared to the median assessor score. This was comparable to the somewhat unreliable inter-rater accuracy observed for the unseen data, which ranged from 57.5 to 67.5%. When the non-linear scale score was converted to a binary impairment/no impairment variable, accuracy improved to 84% against the median assessor score for our 50% network and the recall (88%), precision (84%) and F1 scores (0.88) were all very high. Additionally, there was no statistically significant variance between networks. This is important, because our 50% network (ABI: *n* = 21, HCs: *n* = 17), trained on only half of a relatively small number of patients, can provide data with similar accuracy to human assessment on patients and trials it has not been exposed to previously. Importantly, this allows less time spent training, reducing technical burden, making it feasible to use with location specific training and analysis. However, it is important to note that further research using a higher number of raters than the three experienced ones used in this study may change this finding.

Whilst our study is only proof of concept, our results indicate that applying machine learning for clinical or remote assessment may be greatly beneficial. For example, a telehealth application could assess someone using the available video camera in readily available devices such as a phone, tablet, or computer. After the initial algorithm training is complete, this could be fully automated, without requiring training and validation of the treating clinician’s ability to accurately categorise a patient’s gait impairment in accordance with the subjective scale. A key benefit this provides is that of consistency; there is no variation due to factors such as an individual clinician’s experience or biases, anchoring to other recent assessments or inter-rater variability. The clinicians may still undertake the role of overseeing the test to ensure it is performed correctly, and actioning the results via an intervention plan, but the analysis is automated and performed as if it were examined by a consensus group of experienced assessors. Whether this automation of the assessment leads to clinically meaningful changes in treatment and outcomes requires further study.

Implementing a location specific system, while potentially beneficial, requires some time burden to create. It took approximately one day to label 50% of participants, using one trial for each participant. The networks then took approximately two days of unsupervised training using Tensorflow on a computer with a Nvidia GeForce RTX 2080 Super graphics card. Once this is complete, a server can be setup to automate the data collection, analysis, and reporting. Timing of our analyses revealed that the results could be obtained within one minute of test completion using the current system, which will likely improve with enhanced processing power and more efficient algorithms in the future. With respect to how it could be implemented in a clinical setting, a fixed webcam could be mounted to a wall in the rehabilitation centre, which the clinical assessor uses to record the trial on a phone/tablet/computer. This video could be automatically sent to a local server where the machine learning algorithm can be automated to identify the landmarks, whether via custom trained models such as the one in this study or using the pre-trained models such as Movenet [38]. The server then calculates the rating and sends the results back to the phone/tablet/computer. This can be done with limited privacy issues, as it does not require internet connection. On a larger scale, there is the potential for remote assessment with patients using their smartphone in their own home. This would be a powerful method for community rehabilitation, providing much larger training sets that can be used for specific analyses that encompasses a wider range of environments. However, this has potential privacy issues at present, but future edge-based systems may be able to overcome this.

Our study has limitations. This study was limited by a relatively small sample size (landmark training ABI: *n* = 42, HCs: *n* = 34; subjective score prediction ABI: *n* = 42, HCs: *n* = 5) that necessitated the application of computationally expensive validation (nested cross-validation) to provide a realistic generalisation of the network’s true performance [33]. The sample size may have impacted the network, causing the network to under-predict scores due to the median of scores being heavily distributed around scores 1–2, albeit this was only with small effect size (d = 0.156 to 0.217 across 50%, 75%, 90%, and 100% networks). Despite this, our comparison of participants used in training versus unseen participants had relatively low variances (Fig. 4; Table 2) and similar agreement (Table 3) indicating that our sample size allowed for accurate data to be obtained. Future studies should examine the performance of this methodology with larger sample sizes, where scoring data may be normally distributed amongst both training and test sets. This may enable the training of networks with no statistically significant under- or over-prediction of scores. Additionally, it enables the cross-validation of training models that are less computationally expensive and reflect what would typically be applied in a commercial setting [39], i.e. a simple 80(train):20(test) cross-validation method.

The performance of our machine learning algorithm to label anatomical landmarks may have been impacted by the presence of physical markers on participants that were used for 3D gait analysis in a separate analysis [40, 41]. However, other papers have used this technology without markers to good effect [42, 43]. Kahn, Clark [17] performed their assessment with 3 assessors with > 15 years’ experience, finding that between assessors there was moderate inter-rater reproducibility. Whilst this may have been caused by the application of the scale with limited precision, potentially causing large differences between raters (i.e., between 0 and 1), it is also testament to the difficulty of subjective rating of complex phenomena even when done by highly experienced clinicians. This research may provide a possible solution for the moderate inter-rater reproducibility described by Kahn, Clark [17]. Where assessors experienced scoring variations when exposed to the same phenomenon, potentially due to the ordinal scale (e.g., 0–4), the network would predict an ordinal number independent of the current assessor by applying a composite of previous assessments used to train the network, thereby theoretically eliminating variance between assessors. This could be further studied by having a greater number of assessors results used in the training model, taking advantage of the law of large numbers; however, for this study we prioritised selecting a relatively small number of highly experienced, homogenous assessors over a larger number of more variably experienced assessors.

The study was also limited by the lighting conditions in the room. Lights were controlled via overhead fluorescent lights, however lighting external to the room affected the exposure and subsequent visibility of some joints in a small proportion of the videos. More control for lighting in future studies could increase accuracy of anatomical landmark placement; however, this increased control comes at the cost of less generalisability of the algorithm to different environments. This could be problematic in the event of changes to the environment (for example, switching to newer LED lighting with different spectral bandwidths and luminous ratings). Our data are also not directly generalisable to telehealth, as we controlled the positioning of the camera and maintained consistency of the background and the environment videos were captured in. However, with further network training using data from home environments, it is reasonable to assume that this methodology would be able to accurately assess impairment. However, further studies in home-based settings and different environments are required.

## Conclusion

In conclusion, the restoration of a normal appearance during walking is often an important goal for people with ABI [44, 45]. Clinicians commonly describe abnormal movement deviations in terms of their visual impact and likely subconsciously compare a patient’s presentation to what they expect healthy or “normal” movement patterns to be [46]. Our results indicate that machine learning can quantify this, with similar agreement to experienced clinicians ratings of arm movement abnormality for people living with ABI. This may open the door to more consistent and less biased remote and local assessment. Future research could examine if application of similar systems provides clinical benefits for patients and determine if it is responsive to change amongst larger sample sizes. Future application of this methodology could be applied to the trunk or lower limbs to capture movement abnormality more objectively and simply in clinical settings.

## Data Availability

Raw data for our dataset are not publicly available to preserve individuals’ privacy under the European General Data Protection Regulation. They are however available upon request in an anonymised and supervised form.
